# A Derivate of Benzimidazole-Isoquinolinone Induces SKP2 Transcriptional Inhibition to Exert Anti-Tumor Activity in Glioblastoma Cells

**DOI:** 10.3390/molecules24152722

**Published:** 2019-07-26

**Authors:** He-ying Chen, Liu-jun He, Shi-qiang Li, Ya-jun Zhang, Jiu-hong Huang, Hong-xia Qin, Juan-li Wang, Qian-yin Li, Dong-lin Yang

**Affiliations:** 1 Division of Molecular Nephrology and the Creative Training Center for Undergraduates, the Ministry of Education Key Laboratory of Laboratory Medical Diagnostics, the College of Laboratory Medicine, Chongqing Medical University, Chongqing 400016, China; 2 Chongqing Engineering Laboratory of Targeted and Innovative Therapeutics, Chongqing Key Laboratory of Kinase Modulators as Innovative Medicine, IATTI, Chongqing University of Arts and Sciences, 319 Honghe Ave., Yongchuan, Chongqing 402160, China; 3 The Undergraduates Class of 2016 entry the College of Laboratory Medicine, Chongqing Medical University, Chongqing 400016, China

**Keywords:** compound-7g, glioblastoma, SKP2, E2F-1, cell cycle, autophagic flux

## Abstract

We have previously shown that compound-7g inhibits colorectal cancer cell proliferation and survival by inducing cell cycle arrest and PI3K/AKT/mTOR pathway blockage. However, whether it has the ability to exert antitumor activity in other cancer cells and what is the exact molecular mechanism for its antiproliferation effect remained to be determined. In the present study, compound-7g exhibited strong activity in suppressing proliferation and growth of glioblastoma cells. The inhibitor selectively downregulated F-box protein SKP2 expression and upregulated cell cycle inhibitor p27, and then resulted in G1 cell cycle arrest. Mechanism analysis revealed that compound-7g also provokes the down-regulation of E2F-1, which acts as a transcriptional factor of SKP2. Further results indicated that compound-7g induced an increase of LC3B-II and p62, which causes a suppression of fusion between autophagosome and lysosome. Moreover, compound-7g mediated autophagic flux blockage promoted accumulation of ubiquitinated proteins and then led to endoplasmic reticulum stress. Our study thus demonstrated that pharmacological inactivation of E2F-1-SKP2-p27 axis is a promising target for restricting cancer progression.

## 1. Introduction

Glioblastoma (GBM) presents the most aggressive and common primary malignancy of the central nervous system [1,2]. Although significant advances have been achieved by standard-of-care treatment, GBM remains as an incurable disease due to being the most resistant cancer to radiation and cytotoxic in chemotherapy, therefore triggering an average patient survival rate of approximately 15 months after diagnosis [3,4]. Additionally, it is impossible for surgical treatment to resect all malignant glioma cells, which causes a high recurrence rate [4,5]. In the challenge of developing more effective therapeutic strategies, the acquisition of novel drugs which target proliferating tumor cells but do not harm normal proliferating cells seem to be an imperative strategy for the treatment of patients with GBM.

Recently, S-phase kinase-related protein 2 (SKP2) has been reported as a key cell cycle regulator [6], and has functioned as an oncoprotein in a variety of human cancer cells [7,8]. SKP2 exerts its oncogenic role through recruiting p27 for ubiquitination and subsequent proteasome-dependent degradation, which results in the uncontrolled cell proliferation [9]. E2F-1, a transcription factor, plays a vital role in the control of cellular growth and cell cycle progression. It is reported that *skp2* is a transcriptional target of transcription factor E2F1 [10] and both of them are related to the transition from G1 to S phase. Additionally, E2F and SKP2 highly express in multiple tumor cells in a collaborative manner [11,12,13]. Therefore, identifying compounds that downregulate SKP2 by inhibiting protein content of E2F transcriptional factor might be an effective strategy for cancer therapy such as glioblastoma.

In our previous report, a series of benzimidazole-isoquinolinone derivatives have been synthesized, and compound-7g has been investigated and identified as an excellent inhibitor of colorectal cancer cells. The underlying mechanism of compound-7g mediated anti-tumor activity in colorectal cancer cells was that it provokes G2/M cell cycle arrest, mitochondria-related apoptosis and inhibition of PI3K/AKT/mTOR pathway. Intriguingly, questions remain regarding whether compound-7g has the ability to act as an anti-tumor agent in other types of cancer cells, and whether molecular targets are present which are responsible for the antiproliferative role of compound-7g.

In this current study, we aimed to illustrate the molecular action underlying the anti-tumor effect of compound-7g on glioblastoma cells. We observed that compound-7g induces a decrease of the SKP2 protein level along with increased expression of p27. Interestingly, compound-7g also promoted the E2F-1 degradation to induce transcriptional repression of *skp2.* Further experiments showed that compound-7g provokes G1 cell cycle arrest, autophagic flux blockage and accumulation of ubiquitinated proteins as a result of reduced SKP2 expression. Overall, our data is the first to indicate the involvement of the E2F-1-SKP2-p27 axis in the antitumor action of compound-7g. These results not only provide new insights into the molecular mechanism of compound-7g’s antiproliferative action, but also offer convincing evidence that pharmacological inhibition of *skp2* mRNA level is a promising approach for tumor treatment.

## 2. Results

### 2.1. Compound-7g, as An Effective Anticancer Inhibitor, Can Significantly Inhibit Cell Proliferation and Growth in Glioblastoma Cells

**Figure 1:**
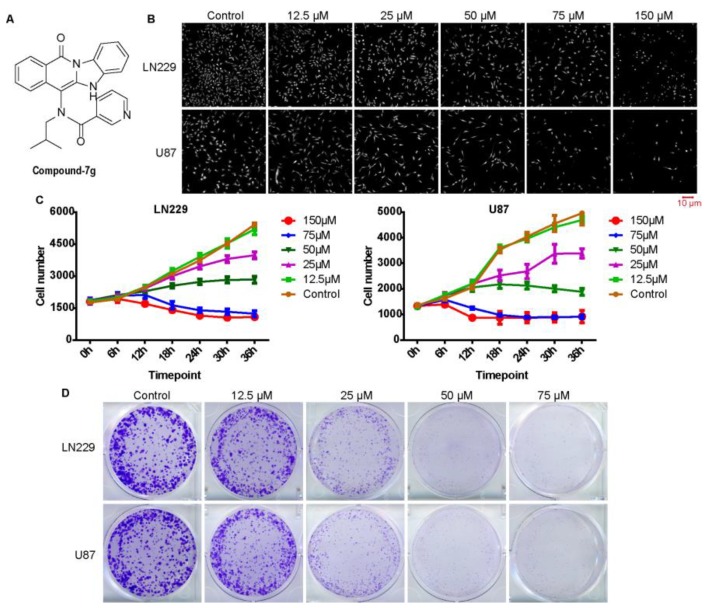
Compound-7g suppresses glioblastoma cells proliferation and viability. (**A**) The chemical structure of compound-7g. (**B**) Digital phase contrast images of LN229 and U87 cells after treatment with compound-7g. Images were captured in high content analysis system-operetta CLSTM. Scale bar, 10 μm. (**C**) Cell growth curve was performed following indicated concentrations of compound-7g treatment. The cell number was analyzed with high content analysis system-operetta CLSTM at an interval of 6h. (**D**) Colony formation assay was performed to evaluate cell growth in vitro after treatment with the indicated concentrations of compound-10# for 14 days. All data were demonstrated as the mean ± SD of three independent experiments.

In the previous study, we found that compound-7g was an effective inhibitor against colorectal cancer cells through impairing PI3K/AKT/mTOR signaling pathway [14]. The structure of compound-7g is shown in Figure 1A. To evaluate whether compound-7g has effect on the most highly aggressive type of brain tumor in adults, cell counting assay with the high content analysis system-operetta CLS™ was performed to assess the role of comound-7g on glioblastoma cell proliferation. The results showed that it induced a dramatically decrease in the total number of cells (Figure 1B). Furthermore, the viability of U87 and LN229 cells was measured with high content analysis system-operetta CLS™ after treatment. The results showed that viability was considerably decreased after treatment with compound-7g in both U87 and LN229 cells (Figure 1C). Additionally, the long-term anti-proliferative efficacy of compound-7g was also determined by a colony formation assay. As shown in Figure 1D, in comparison with control groups, smaller and lesser colonies were formed after cells exposing to 25 μM compound-7g, and rarely no colony formed when treating with 50 and 75 μM, which were consistent with cell viability data, suggesting its strong capability in reducing glioblastoma cell proliferation and growth. Collectively, these results supported that compound-7g is an effective inhibitor which has high in vitro antiproliferative ability in glioblastoma cells.

### 2.2. Compound-7g Induces Transcriptional Repression of Skp2 By Promoting E2F-1 Degradation

**Figure 2:**
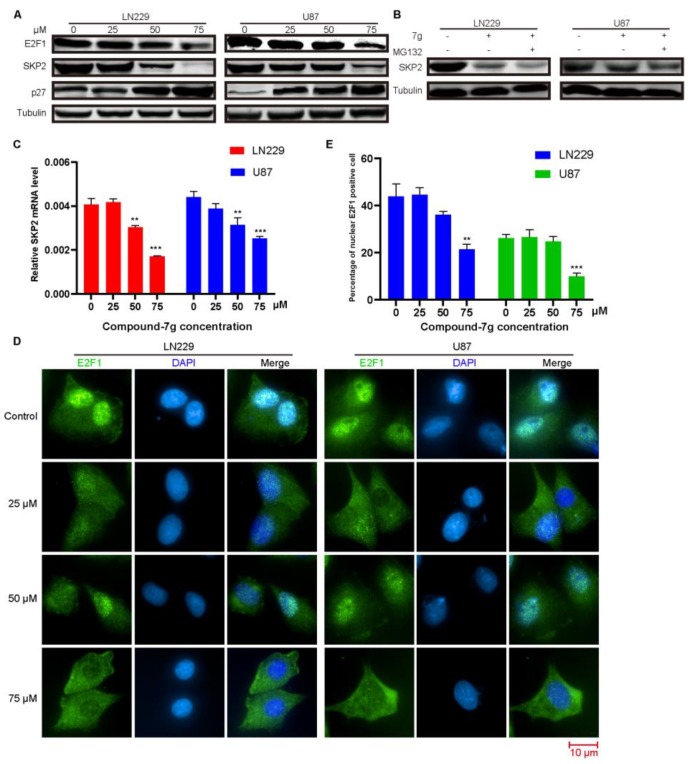
Compound-7g induces transcriptional repression of *skp2* by promoting E2F-1 degradation. (**A**) Immunoblotting was explored to evaluate the expression of SKP2, p27 and E2F-1 after treatment with compound-7g in LN229 and U87 cells. (**B**) Immunoblotting was performed to detect the effect of combination between compound-7g and MG132 on SKP2 expression. (**C**) The mRNA expression level of *skp2* was analyzed by real-time PCR. Actin was used as an internal control in the mRNA analysis experiments. (**D**) Immunofluorescence was performed to further detect the E2F-1 expression as well as localization change. The images were captured by high content analysis system-operetta CLS™. Scale bar: 10 μm. (**E**) Histograms represent the percentages of nuclear E2F-1 positive cells in (**D**). The data are shown as the means ± SD (n = 3). * *P* < 0.05; ** *P* < 0.01; *** *P* < 0.001 compared with the control group.

To explore the anti-tumor mechanism underlying antiproliferative activity of the compound-7g in human glioblastoma cells, the expression level of E3 ubiquitin ligase SKP2 was detected by immunoblotting in both U87 and LN229 cells after treatment with the inhibitor. As shown in Figure 2A, the SKP2 expression level was significantly down-regulated in a dose-dependent manner. Furthermore, we investigated the affection of compound-7g on p27, which is a downstream substrate of SKP2, and also promoted ubiquitination and degradation by SKP2. Consistent with our hypothesis, p27 expression was dramatically increased in a dose dependent manner, suggesting that compound-7g inhibits its degradation by affecting SKP2 expression. We next evaluated the mechanism responsible for compound-7g induced SKP2 degradation. To address this issue, we investigated the consequence of combination compound-7g with MG132 treatment on SKP 2 expression level or the mRNA level of skp2 after exposure to compound-7g. As indicated in Figure 2B,C, a dose-dependent decrease in *skp2* mRNA level was detected following compound-7g treatment, while SKP2 protein level was still effectively reduced by compound-7g treatment regardless of MG132 addition, implying that compound-7g promotes SKP2 decrease by transcriptional repression. Given that SKP2 is a transcriptional target of transcription factor E2F1 [10], we were interested in determining whether it is involved in this effect induced by compound-7g. We found that compound-7g dose-dependently lowered the level of E2F-1 (Figure 2A). Consistently, immunofluorescence analysis exerted a prominent decrease of the E2F-1 protein expression level, as verified by the high accumulation of green signal localized in the nucleus of control cells that was nearly absent in cells treated with compound-7g, suggesting the degradation and delocalization of E2F-1 after treatment (Figure 2D,E). Collectively, these results demonstrated that compound-7g induced SKP2 down-regulation and p27 up-regulation is likely to be caused by E2F-1 degradation induced transcriptional repression.

### 2.3. Compound-7g Induces G1-Phase Arrest of Glioblastoma Cells

**Figure 3:**
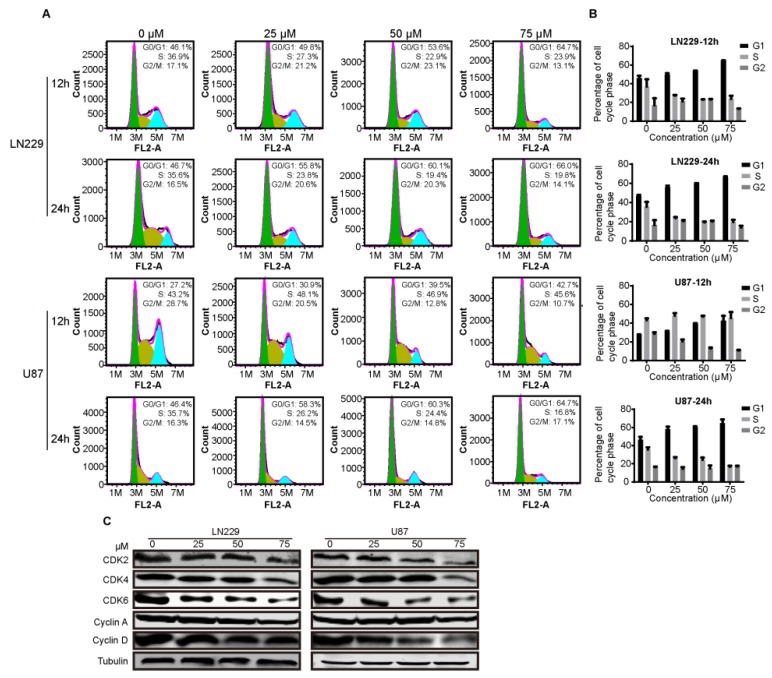
Compound-7g results in the G1 cell cycle arrest in glioblastoma cells. (**A**,**B**) The cell cycle of LN229 and U87 cells was measured by flow cytometry in the presence of compound-7g and the percentages of cell population in different periods were quantitated with three independent experiments. (**C**) Impact of compound-7g on the expression levels of G1 phase related proteins in LN229 and U87cells was determined by immunoblotting assay. Tubulin was used as a loading control.

Given that SKP2 is an E3 ligase to recruit p27 for ubiquitination and subsequent proteasome degradation, and functions as a positive regulator of the cell cycle by promoting S-phase entry [9,15], we were interested in determining whether compound-7g could serve as an inhibitor to induce G1-phase arrest by promoting SKP2 down-regulation. To this end, cells were exposed to compound-7g for 12 h and 24 h, respectively, and the cell cycle in treated cells was arrested at G1 phase by flow cytometry analysis (Figure 3A,B). Furthermore, to confirm the results, immunoblotting was explored to determine the protein levels involved in the G1 phase, and we found that compound-7g treatment reduced the levels of Cyclin A, Cyclin E, CDK2, CDK4 and CDK6 in a dose-dependent manner (Figure 3C). Taken together, these results demonstrated that compound-7g treatment results in G1-phase cell cycle arrest because of SKP2 down-regulation and p27 up-regulation.

### 2.4. Compound-7g Inhibits the Fusion Between Autophagosome And Lysosome in Glioblastoma Cells

**Figure 4:**
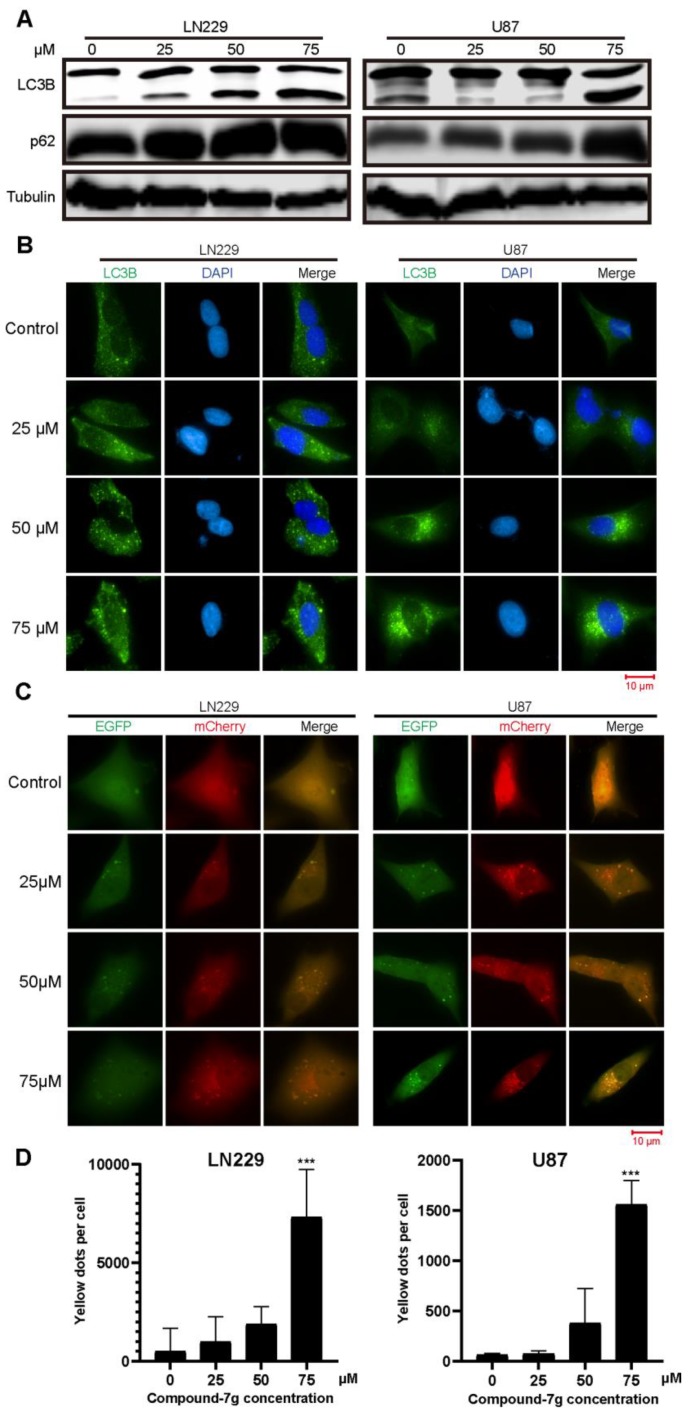
Compound-7g inhibits the autophagic flux in both LN229 and U87 cells. (**A**) LC3B-II and p62 were detected using immunoblotting to evaluate the effect of compound-7g on autophagy. (**B**) Immunofluorescence was performed to analyze LC3B after exposing to the indicated concentrations of compound-7g. Scale bar: 10 μm. (**C**) Both LN229 and U87 cells were stably transfected with mCherry-EGFP-LC3B. Yellow fluorescence indicates the formation of autophagosomes while the red signal represents the acidic autophagolysosomes. Scale bar, 10 μm. (**D**) Quantitative analysis of the autophagosome accumulation effect of compound-7g-treated cells. Yellow dots were counted in three independent experiments shown in (**D**). The data are expressed as means ± SD (n = 3). * *P* < 0.05; ** *P* < 0.01; *** *P* < 0.001 compared with the control group.

Since SKP2 has been reported to be involved in the autophagy [16,17,18], we tested whether compound-7g can play a role in the autophagy through regulating expression levels of LC3B-II and p62, two specific and conserved markers of autophagy representing autophagosome formation or progression of autophagic flux. As shown in Figure 4A, immunoblotting revealed that conversion of non-lipidated LC3B-I to lipidated LC3B-II and p62 were significantly increased in a dose-dependent manner following compound-7g treatment but were not in control, suggesting that the autophagic flux is blocked because the augmentation of LC3B-II and p62 symbolizes augophagosomes accumulation. To further confirm this result, immunofluorescence assay was performed following U87 and LN229 exposure to various concentrations of compound-7g. The green punctum representing intrinsic LC3B signal mainly localized in the cytoplasm and was notably increased in compound-7g treated cells compared to control group (Figure 4B). Furthermore, we interested in whether compound-7g can inhibit the fusion between autophagosome and lysosome which induces blockage of autophagic flux, a PH sensitive, double tagged mCherry-GFP-LC3B reporter was used to evaluate the fusion efficiency. A notable accumulation of yellow fluorescent vesicles in compound-7g treated cells as compared with control was observed, implying that an increase of autophagosomes is induced by a deficiency of fusion between autophagosome and lysosome (Figure 4C). Consistently, the histogram in Figure 4D demonstrated that the yellow puncta representing the number of non-acidic autophagosomes was increased in a dose-dependent manner. Taken together, these results indicated that compound-7g mediated accumulation of LC3-II and p62 is dependent on the inhibition of fusion between autophagosome and lysosome.

### 2.5. Compound-7g Induced Autophagic Flux Blockage Results in Accumulation of Ubiquitinated Proteins and Subsequent ER Stress

**Figure 5:**
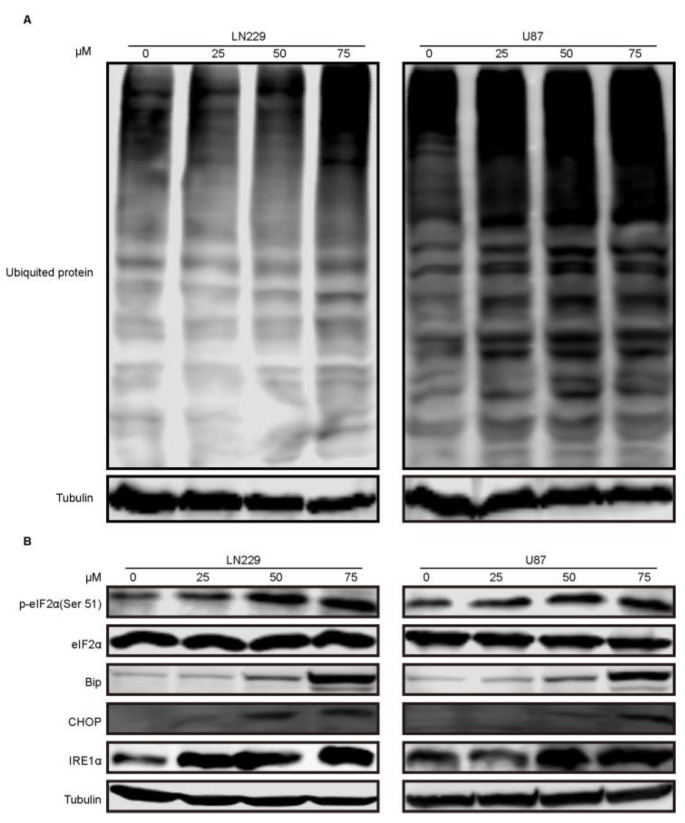
Compound-7g promoted accumulation of ubiquitinated proteins and ER stress because of induced autophagic flux blockage in LN229 and U87 cells. (**A**,**B**) Immunoblotting analysis for the ubiquitinated proteins and expression of ER stress related proteins in glioblastoma cells treated with the indicated concentrations of compound-7g. Tubulin was loaded as a control.

Given that inhibition of autophagic flux could result in accumulation of ubiquitinated proteins, we examined whether compound-7g induced blockage of fusion between autophagosome and lysosome can lead to the decrease of protein aggregate clearance. As shown in Figure 5A, immunoblotting indicated that ubiquitinated aggregates were accumulated in a dose-dependent manner following treatment with compound-7g. Since previous studies reported that elevated ubiquitinated proteins could induce ER stress, we tested the effect of accumulated intracellular misfolded proteins on ER stress. Immunoblotting result revealed that ER stress related proteins such as phosphorylation-eIF2α (Ser51), Bip, CHOP and IRE1α were increased in a dose-dependent manner after exposure to compound-7g, implying that accumulation of unfolded or misfolded proteins induced by the inhibitor significantly enhances ER stress (Figure 5B). Therefore, these observations demonstrated that accumulation of ubiquitinated proteins induced by compound-7g promotes ER stress.

## 3. Discussion

A number of compounds with benzimidazole scaffolds can lead to cell-cycle arrest or provoke apoptosis by regulating kinase signal pathway. Our previous study showed that compound-7g exerts the most promising activity against CRC lines [14]. In our current study, we found compound-7g exhibits significant antitumor activity in both U87 and LN229 glioblastoma cells, implying that compound-7g may be a broad-spectrum inhibitor. During the process of exploring the potential mechanism of compound-7g induced antiproliferative activity, we proved that compound-7g promotes G1 cell cycle arrest in glioblastoma cells compared to G2/M cell cycle blockage in colorectal cancer cells. We speculate that this difference of cell cycle arrest results from the diverse tumor location such as colorectal cancer, called digestive system tumor, and glioblastoma, called brain tumor, and different pathological mechanism.

Increasing reports have demonstrated that SKP2 functions as an oncoprotein in tumorigenesis and overexpression of SKP2 is related to poor prognosis [19,20]. Notably, SKP2 exhibits its physiological role through ubiquitination and subsequent degradation of its substrates such as p21 [21], p27 [22], p57 [23], p53 [24] and Foxo1 [25]. Furthermore, cell cycle, especially G1/S phase, is blocked because of the degradation of CDK inhibitors, and then cell proliferation is enhanced with a SKP2-dependent manner. In line with these, our previous research showed that compound-7g leads to the upregulation of p21 and p53 and G2/M cell cycle arrest in colorectal cancer cells [14]. In this study, our results indicated that compound-7g suppresses cell cycle at G1 phase by promoting SKP2 decrease and p27 accumulation, suggesting that SKP2 induced p27 degradation is inhibited by compound-7g in glioblastoma cells. Thus, compound-7g may function as an inhibitor in both CRC and glioblastoma cells by down-regulating SKP2.

The deficiency of autophagic flux results in the accumulation of cytotoxic misfolded protein aggregates which induce the cell death [26,27]. Recently, several reports demonstrated that SKP2 is involved in autophagy activation for promoting cancer cell proliferation and survival [17,18]. Moreover, under nutrient starvation condition, transcriptional repression of SKP2 was induced by AMP-activated protein kinase (AMPK)-dependent phosphorylation of FOXO3a in the nucleus. Its suppression promoted up-regulated co-activator-associated arginine methyltransferase 1 (CARM1), which in turn initiated autophagy because CARM1 functions as a transcriptional co-activator on autophagy-related and lysosomal genes through transcription factor EB (TFEB) [28]. However, the function of SKP2 in the process of autophagic flux such as fusion between autophagosome and lysosome is unclear so far. We discovered that compound-7g induced accumulation of LC3B-II and p62 is dependent on the inhibition of fusion between autophagosome and lysosome, implying that an SKP2 decrease might relate to regulation of autophagic flux. To the best of our knowledge, this is the first study demonstrating that SKP2 is involved in the fusion between autophagosome and lysosome.

## 4. Materials and Methods

### 4.1. Antibodies and Reagents

The following antibodies which were used in this study: anti-Tubulin, anti-CDK2, anti-CDK4, anti-CDK6, anti-Cyclin A, anti-Cyclin D, anti-E2F1, anti-SKP2, anti-LC3B, anti-p62, anti-eIF2α, anti-p-eIF2α(S51), anti-BIP, anti-CHOP, anti-IRE1α were purchased from Cell Signaling Technology. The following chemical reagents which were used in this study: DAPI, PI and DMSO were purchased from sigma, MG132 and Chloroquine (CQ) were purchased from Selleck.

### 4.2. Cell Lines and Culture

Human glioblastoma cell lines U87 and LN229 were purchased from the American Type Culture Collection (ATCC, Manassas, VA, USA). U87 and LN229 were maintained in high-glucose DMEM (Hyclone, SH30022.01, USA) a medium was added of 10% fetal bovine serum (FBS, Gibco, 10100147, Australia origin) and it was cultured in an incubator with 37 °C and 5% CO_2_ with humidified atmosphere.

### 4.3. Cell Viability Analysis

U87 and LN229 cells were harvested in the density of 80% and seeded into the 96-well plate with density of 2 × 10^3^ cells per well containing 100 μL complete medium. After incubation for 24 h, we added another 100 μL complete medium containing compound-7 g with variant concentrations (0, 12.5, 25, 50, 75, 150 μM), and put it into the High Content analysis system (Perkinelmer, Waltham, MA, USA) for incubation. Afterwards, cell viability was analyzed using it.

### 4.4. Colony Formation Assay

U87 and LN229 were seeded in the 6-well plate with density of 500 cells per well containing 1 mL complete medium. After incubation 24 h, treated with compound-7 g at concentration of 0, 12.5, 25, 50 and 100 μM. For another 14 days of incubation, cells were fixed with 4.0% paraformaldehyde for 25 min and stained with 0.5% crystal violet.

### 4.5. Immunoblotting

Cells were harvested and then resuspended the cells in RIPA buffer (Beyotime, Shanghai, China) supplemented with protease and phosphatases inhibitors (Roche, 4906837001, Mannheim, Germany). The samples were subjected to sodium dodecyl sulfate polyacrylamide gelelectrophoresis (SDS-PAGE) gels and then transferred onto polyvinylidene difluoride (PVDF) membranes (Millipore Corporation, ISEQ00010, Billerica, MA, USA). The membranes were experienced incubation with QuickBlock™ Blocking Buffer (Beyotime, P0228, Shanghai, China), primary antibodies, and IRDye 800CW goat anti-mouse IgG (H + L) or IRDye 680LT donkey anti-rabbit IgG (H + L) (Li-cor, Lincoln, NE, USA) secondary antibody. Immunoreactivity was detected by an Odyssey two-color infrared fluorescence imaging system (Li-cor, Lincoln, NE, USA).

### 4.6. Cell Cycle Measurement

Cells were treated with compound-7g at indicated concentrations (0, 25, 50, 75 μM) for 12 h and 24 h, respectively. Cells were harvested and fixed in 70% ethanol for 12 h at 4 °C. Before staining, the cells were resuspended in the PBS supplemented with 50 μg/mL propidium iodide (PI, Sigma, P4170, MO, USA) and RNase A (Beyotime, ST579, Shanghai, China), and then analyzed by flow cytometry (Becton Dickinson, San Jose, CA, USA).

### 4.7. RNA Extraction and Real-Time Fluorescent Quantitative PCR

The total RNA of LN229 and U87 cells treated with compound-7g were extracted using the RNAprep pure Cell/Bacteria Kit (TIANGEN, DP430, Beijing, China). The reverse transcription of 2 μg total RNA sample was performed by the PrimScript™ RT Master Mix (perfect real time) (Takara, RR036A, Tokyo, Japan). The abundance of mRNA was measured by the LightCycler96 (Roche, Mannheim, Germany) using the TB Green Fast qPCR Mix (Takara, RR091A, Tokyo, Japan). The *ACTB* was used as a housekeeping gene.

### 4.8. Immunofluorescence Staining

LN229 and U87 cells were seeded in the CellCarrier™-96-well plate (PerkinElmer, Waltham, MA, USA) at a density of 5 × 10^3^ cells per well. After being incubated for 24 h, cells were treated with compound-7g at various concentrations. Cells were fixed with 4% paraformaldehyde for 25 min at room temperature, and then blocked for 30 min at 37 °C using QuickBlock™ Blocking Buffer for Immunol Staining (Beyotime, P0260, Shanghai, China). The cells were incubated with anti-E2F1 or anti-LC3B for 2 h at room temperature. Then, cells were incubated with Alexa Fluor 488-conjugated anti-rabbit antibody for 1 h (Thermo, A32731, Waltham, MA, USA) at room temperature, and then incubated with DAPI for 15 min at room temperature. The samples were analyzed by the High Content analysis system.

### 4.9. Fluorescence Observation of Mcherry-GFP-LC3B

It is reported that double tagged mCherry-GFP-LC3B reporter, a pH-sensitive plasmid, could effectively determine the fusion efficiency between autophagosome and lysosome [29,30]. Hence, LN229 and a U87 cell stable expressing mCherry-GFP-LC3B (YouBio, V2155, Changsha, China) were constructed using a lentivirus infection process. The mCherry-GFP-LC3B, Pspax2 and pMD2G vectors were co-transfected into the HEK293T cells using the Lipo8000™ Transfection Reagent (Beyotime, C0533, Shanghai, China). After incubatation for 72 h, the virus particles were collected, and then added into the LN229 and U87 cells medium supplemented with 10 μg/mL polybrene. The medium was replaced after incubating 24 h and then selected by 10 μg/mL puromycin to obtain the cells which stable express the mCherry-GFP-LC3B vector. The transgenic cells were treated with the compound-7g and analyzed using the high content analysis system-operetta CLS™ (PerkinElmer, Waltham, MA, USA).

### 4.10. Statistical Analysis

All data was analyzed with GraphPad Prism 8.0 and showed using the mean ± standard deviation (SD). Statistical significance of the data was determined by the two-tailed independent Student’s *t*-test, and a *p* < 0.05 was considered statistically significant. Each experiment was performed in triplicate.

## 5. Conclusions

The findings presented in this report indicate that compound-7g promotes G1 cycle arrest and autophagic flux blockage by down-regulating SKP2 expression. Furthermore, suppression of fusion between autophagosome and lysosome results in the accumulation of ubiquitinated protein and ER stress. Mechanistically, compound-7g induces E2F-1 degradation to transcriptionally down-regulate SKP2. Therefore, our study not only provides new insights into the molecular mechanism of compound-7g’s antiproliferative action, but also offers convincing evidence that pharmacological inactivation of E2F-1-SKP2-p27 axis is a promising approach for tumor treatment.

## References

[1] Stupp R., Mason W.P., Van Den Bent M.J., Weller M., Fisher B., Taphoorn M.J., Belanger K., Brandes A.A., Marosi C., Bogdahn U. (2005). Radiotherapy plus concomitant and adjuvant temozolomide for glioblastoma. N. Engl. J. Med..

[2] Wen P.Y., Kesari S. (2008). Malignant gliomas in adults. N. Engl. J. Med..

[3] Sathornsumetee S., Reardon D.A., Desjardins A., Quinn J.A., Vredenburgh J.J., Rich J.N. (2007). Molecularly targeted therapy for malignant glioma. Cancer.

[4] Hueng D.-Y., Hsieh C.-H., Cheng Y.-C., Tsai W.-C., Chen Y. (2017). Cordycepin inhibits migration of human glioblastoma cells by affecting lysosomal degradation and protein phosphatase activation. J. Nutr. Biochem..

[5] Liu Y., Fan C., Pu L., Wei C., Jin H., Teng Y., Zhao M., Yu A.C.H., Jiang F., Shu J. (2016). Phloretin induces cell cycle arrest and apoptosis of human glioblastoma cells through the generation of reactive oxygen species. J. Neuro-Oncol..

[6] Lee Y., Lim H.-S. (2016). Skp2 inhibitors: Novel anticancer strategies. Curr. Med. Chem..

[7] Wang Z., Liu P., Inuzuka H., Wei W. (2014). Roles of F-box proteins in cancer. Nat. Rev. Cancer.

[8] Chan C.-H., Morrow J.K., Zhang S., Lin H.-K. (2014). Skp2: A dream target in the coming age of cancer therapy. Cell Cycle.

[9] Chu I.M., Hengst L., Slingerland J.M. (2008). The Cdk inhibitor p27 in human cancer: Prognostic potential and relevance to anticancer therapy. Nat. Rev. Cancer.

[10] Zhang L., Wang C. (2006). F-box protein Skp2: A novel transcriptional target of E2F. Oncogene.

[11] Reichert M., Saur D., Hamacher R., Schmid R.M., Schneider G. (2007). Phosphoinositide-3-kinase signaling controls S-phase kinase–associated protein 2 transcription via E2F1 in pancreatic ductal adenocarcinoma cells. Cancer Res..

[12] Lu Z., Bauzon F., Fu H., Cui J., Zhao H., Nakayama K., Nakayama K.I., Zhu L. (2014). Skp2 suppresses apoptosis in Rb1-deficient tumours by limiting E2F1 activity. Nat. Commun..

[13] Salon C., Merdzhanova G., Brambilla C., Brambilla E., Gazzeri S., Eymin B. (2007). E2F-1, Skp2 and cyclin E oncoproteins are upregulated and directly correlated in high-grade neuroendocrine lung tumors. Oncogene.

[14] He L.-J., Yang D.-L., Li S.-Q., Zhang Y.-J., Tang Y., Lei J., Frett B., Lin H.-K., Li H.-Y., Chen Z.-Z. (2018). Facile construction of fused benzimidazole-isoquinolinones that induce cell-cycle arrest and apoptosis in colorectal cancer cells. Bioorgan Med. Chem..

[15] Kurland J.F., Tansey W.P. (2004). Crashing waves of destruction: The cell cycle and APCCdh1 regulation of SCFSkp2. Cancer Cell.

[16] Wu H., Wang Y., Wang X., Li R., Yin D. (2017). MicroRNA-365 accelerates cardiac hypertrophy by inhibiting autophagy via the modulation of Skp2 expression. Biochem. Biophys. Res. Commun..

[17] Jung D., Khurana A., Roy D., Kalogera E., Bakkum-Gamez J., Chien J., Shridhar V. (2018). Quinacrine upregulates p21/p27 independent of p53 through autophagy-mediated downregulation of p62-Skp2 axis in ovarian cancer. Sci. Rep..

[18] Liu K., Zhang L., Zhao Q., Zhao Z., Zhi F., Qin Y., Cui J. (2018). SKP2 attenuates NF-κ B signaling by mediating IKK β degradation through autophagy. J. Mol. Cell Boil..

[19] Hao Z., Huang S. (2015). E3 ubiquitin ligase Skp2 as an attractive target in cancer therapy. Front. Biosci. (Landmark Ed.).

[20] Lu W., Liu S., Li B., Xie Y., Adhiambo C., Yang Q., Ballard B.R., Nakayama K.I., Matusik R.J., Chen Z. (2015). SKP2 inactivation suppresses prostate tumorigenesis by mediating JARID1B ubiquitination. Oncotarget.

[21] Wei Z., Jiang X., Qiao H., Zhai B., Zhang L., Zhang Q., Wu Y., Jiang H., Sun X. (2013). STAT3 interacts with Skp2/p27/p21 pathway to regulate the motility and invasion of gastric cancer cells. Cell. Signal..

[22] Luo J., Zhou Y., Wang B., Li Q., Chen Y., Lan H. (2015). Immunohistochemically detected expression of Skp2, p27 kip1, and p-p27 (Thr187) in patients with cholangiocarcinoma. Tumor. Biol..

[23] Yang C., Nan H., Ma J., Jiang L., Guo Q., Han L., Zhang Y., Nan K., Guo H. (2015). High Skp2/low p57Kip2 expression is associated with poor prognosis in human breast carcinoma. Breast Cancer Basic Clin. Res..

[24] Yeh C.T., So M., Ng J., Yang H.W., Chang M.L., Lai M.W., Chen T.C., Lin C.Y., Yeh T.S., Lee W.C. (2010). Hepatitis B virus–DNA level and basal core promoter A1762T/G1764A mutation in liver tissue independently predict postoperative survival in hepatocellular carcinoma. Hepatology.

[25] Wang H., Cui J., Bauzon F., Zhu L. (2010). A comparison between Skp2 and FOXO1 for their cytoplasmic localization by Akt1. Cell Cycle.

[26] Beth L., Guido K. (2008). Autophagy in the pathogenesis of disease. Cell.

[27] Kawaguchi Y., Kovacs J.J., McLaurin A., Vance J.M., Ito A., Yao T.-P. (2003). The deacetylase HDAC6 regulates aggresome formation and cell viability in response to misfolded protein stress. Cell.

[28] Shin H.-J.R., Kim H., Oh S., Lee J.-G., Kee M., Ko H.-J., Kweon M.-N., Won K.-J., Baek S.H. (2016). AMPK–SKP2–CARM1 signalling cascade in transcriptional regulation of autophagy. Nature.

[29] Lee J.Y., Koga H., Kawaguchi Y., Tang W., Wong E., Gao Y.S., Pandey U.B., Kaushik S., Tresse E., Lu J. (2010). HDAC6 controls autophagosome maturation essential for ubiquitin-selective quality-control autophagy. EMBO J..

[30] Mizushima N., Yoshimori T., Levine B. (2010). Methods in mammalian autophagy research. Cell.

